# Activation of autophagic flux by epigallocatechin gallate mitigates TRAIL-induced tumor cell apoptosis via down-regulation of death receptors

**DOI:** 10.18632/oncotarget.11597

**Published:** 2016-08-25

**Authors:** Sung-Wook Kim, Ji-Hong Moon, Sang-Youel Park

**Affiliations:** ^1^ Biosafety Research Institute, Department of Veterinary Medicine, College of Veterinary Medicine, Chonbuk National University, Iksan, Jeonbuk 54596, Republic of Korea

**Keywords:** EGCG, autophagy, TRAIL, death receptor

## Abstract

Epigallocatechin gallate (EGCG) is a major polyphenol in green tea. Recent studies have reported that EGCG can inhibit TRAIL-induced apoptosis and activate autophagic flux in cancer cells. However, the mechanism behind these processes is unclear. The present study found that EGCG prevents tumor cell death by antagonizing the TRAIL pathway and activating autophagy flux. Our results indicate that EGCG dose-dependently inhibits TRAIL-induced apoptosis and decreases the binding of death receptor 4 and 5 (DR4 and 5) to TRAIL. In addition, EGCG activates autophagy flux, which is involved in the inhibition of TRAIL cell death. We confirmed that the protective effect of EGCG can be reversed using genetic and pharmacological tools through re-sensitization to TRAIL. The inhibition of autophagy flux affects not only the re-sensitization of tumor cells to TRAIL, but also the restoration of death receptor proteins. This study demonstrates that EGCG inhibits TRAIL-induced apoptosis through the manipulation of autophagic flux and subsequent decrease in number of death receptors. On the basis of these results, we suggest further consideration of the use of autophagy activators such as EGCG in combination anti-tumor therapy with TRAIL.

## INTRODUCTION

Tumor necrosis factor-related apoptosis-inducing ligand (TRAIL) is a promising anti-cancer therapeutic agent that can induce apoptotic cell death [1–3]. Recent studies have found that TRAIL can bind to decoy receptors (1 and 2) and inhibit the activation of apoptosis [4]. TRAIL also subsequently recruits FADD and activates caspase-8, thus inducing the apoptotic pathway [5]. An increase in death receptor (DR) protein levels is associated with survival in patients with colon cancer [6, 7]. TRAIL has been shown to induce apoptotic cell death in various cancer cells by binding to death receptor 4 and 5 (DR4 and 5) and activating the extrinsic apoptosis pathway [8]. However, most normal cells are relatively resistant to TRAIL treatment [9, 10], indicating that TRAIL can selectively induce apoptotic cell death after activating death receptors.

Epigallocatechin gallate (EGCG) is the most abundant polyphenol in green tea. Major green tea catechins such as epigallocatechin (EGC; 3–6%), epicatechin-3-gallate (ECG; 3–6%), epicatechin (EC; 1–3%), and catechins (C; less than 1%) have been used both in the human diet and topical therapy for several pathological conditions related to oxidative stress or UVB radiation [11, 12]. EGCG can induce apoptotic cell death, and thus inhibit the migration and invasion of human cervical cancer cells [13]. In addition, EGCG can inhibit the proliferation of glioma cells and decrease their invasive activities [14]. EGCG has been found to induce ROS generation and activate p38 MAP kinase in human endometrial adenocarcinoma cells [15]. However, recent studies have also found that EGCG can activate autophagic flux in many types of cells (including cancer cells) and protect neurodegeneration in primary neuron cells [16–18]. These results have demonstrated that EGCG plays a role in the protection of a variety of cells involved in autophagy activation.

The activation of autophagic flux can inhibit TRAIL-induced apoptosis in TRAIL-resistant cells [19, 20]. Autophagy, known as the lysosomal degradation process, can induce cell death and cell survival [21]. Autophagy plays an important role in cellular homeostasis by deleting long-lived proteins, misfolded proteins, and intracellular organelles. Immoderate autophagy can induce cellular destruction [22, 23]. In addition, autophagic flux can prevent cell death during hypoxia, starvation, growth factor deprivation, endoplasmic reticulum (ER) stress, and microbial infection [24]. However, activation of autophagic flux is also associated with cell death resulting from caspase activation, lysosomal membrane permeabilization, and dysfunction of mitochondrial membrane potential [25]. Autophagic cell death is induced by the accumulation of autophagic organelles, including autophagosomes and autophagolysosomes, in neurodegenerative diseases [22, 23, 26]. P62/SQSTM1 plays an important role as an autophagy marker in the degradation of polyubiquitinated substrates by autophagy flux, thus causing its own degradation [27]. It has been demonstrated that TRAIL-resistant cells exhibit high autophagic flux with increased clearance of p62 protein, while TRAIL-sensitive cells has low autophagic flux and accumulation of p62 [28]. Moreover, p62 protein can bind to DISC and enhance the activation, aggregation, and processing of caspase-8, a known pro-apoptotic factor [29]. The objective of this study was to determine whether EGCG treatment could down-regulate the protein levels of death receptors and activate autophagy flux in human colon cancer cells. Our results showed that EGCG-induced autophagy flux activation inhibits TRAIL-induced apoptosis by regulating DR5.

## RESULTS

### EGCG protected TRAIL-induced apoptosis in HCT116 cells

**Figure 1:**
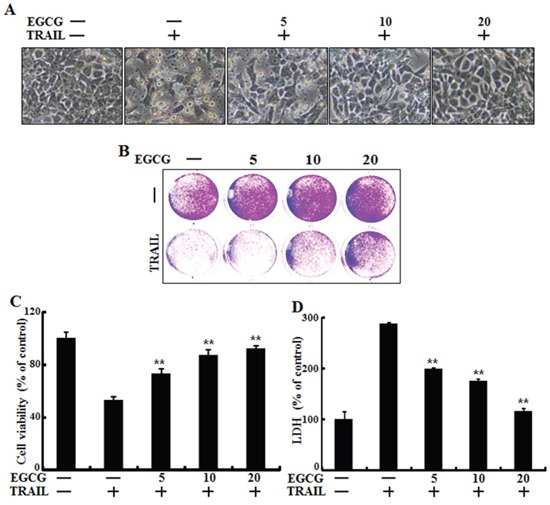
EGCG protected TRAIL-induced apoptosis in HCT116 cells HCT116 cells were pretreated with EGCG (5-20 μM) for 12 h followed by incubation with recombinant TRAIL (100 ng/ml) for an additional 2 h. **A.** Cells were photographed under light microscopy (×200); **B, C.** Viable cells were stained with crystal violet. The viability of control cells was set at 100%. The viability relative to the control was estimated. Results were representatives of three independent experiments; **D.** LDH released into cell culture supernatants. **p < 0.01 indicating significant differences between control group and treatment group.

To determine the effect of EGCG treatment on TRAIL-induced apoptosis in HCT116 human colorectal cancer cells, changes in cell morphology and viability were monitored using light microscopy, crystal violet assay, and LDH release assay. After the cells were pretreated with the indicated doses of EGCG for 12 h, they were treated with TRAIL for an additional 2 h. TRAIL treatment markedly increased cell death, indicating that HCT116 cells were sensitive to TRAIL treatment. Additionally, EGCG inhibited TRAIL-induced cell death in a dose-dependent manner. Cell morphology data showed that the combination of TRAIL and EGCG decreased the number of apoptotic cells compared to TRAIL treatment alone (Figure 1A). EGCG and TRAIL co-treatment increased cell viability and decreased LDH release (Figure 1B-1D). Overall, these data indicate that EGCG treatment inhibits TRAIL-induced apoptosis in HCT116 human colon cancer cells.

### EGCG decreased the protein levels of death receptors

**Figure 2:**
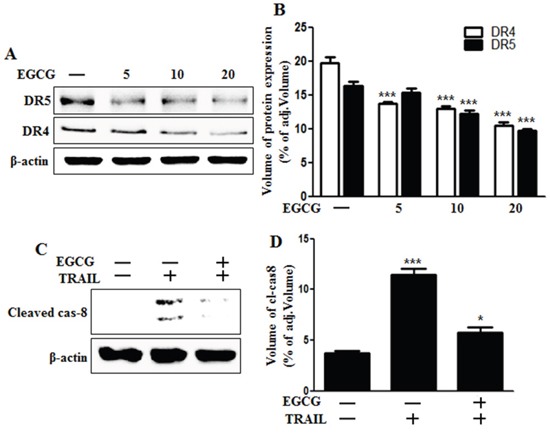
EGCG decreased the protein levels of death receptors **A, B.** HCT116 cells were treated with EGCG at 5-20 μM for 12 h and subjected to western blot analysis for DR4 and DR5 proteins. β-actin was used as loading control; **C.** Whole cell lysates were subjected to western blot analysis for cleaved caspase-8. β-actin was used as loading control. **D.** Bar graph indicating the averages of cleaved caspase-8 protein levels. *** *p* < 0.001: Significant differences between the control group and treatment group.

Recent studies have reported that TRAIL can bind with specific receptors such as DR4 (also known as TRAIL-R1) and DR5 (also known as TRAIL-R2) [4, 30]. To examine the effect of EGCG on TRAIL-related death receptor protein levels, we treated HCT116 cells with different doses of EGCG for 12 h. Western blot analysis showed that DR4 and 5 protein levels were decreased after treatment with EGCG in a dose-dependent manner (Figure 2A and 2B). As shown in Figure 2C and 2D, EGCG and TRAIL co-treatment decreased the production of the activated form of apoptotic factors, such as caspase-8, as compared to treatment with TRAIL alone.

### EGCG stimulated autophagic flux

**Figure 3:**
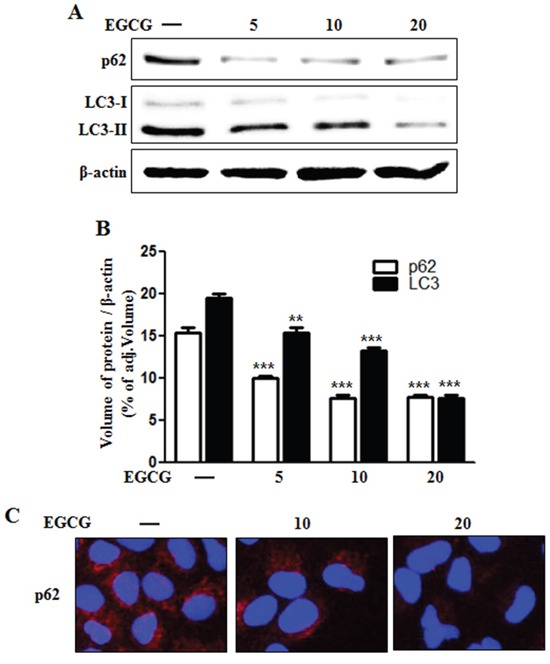
EGCG stimulated autophagy flux **A.** HCT116 cells were treated with EGCG at 5-20 μM for 12 h and subjected to western blot analysis for p62, LC3-I, and LC3-II proteins. β-actin was used as loading control; **B.** Bar graph indicating the averages of p62 and LC3-II protein levels. **C.** Representative images of p62 protein in HCT116 cells. **p < 0.01, *** *p* < 0.001: Significant differences between the control group and treatment group.

Some studies have implicated autophagic flux in the activation of apoptotic signaling factors such as cleaved caspase-3 and cleaved caspase-8 in TRAIL-induced apoptosis [29, 31, 32]. Therefore, we evaluated the induction of autophagic flux markers such as microtubule-associated light chain 3 (LC3) and p62 proteins using western blot analysis and immunofluorescence staining. Western blot analysis revealed that the protein level of both p62 and LC3-II was decreased by EGCG treatment in a dose-dependent manner (Figure 3A and 3B). During the autophagy process, LC3-I is converted into its autophagosomal membrane form LC3-II, which is the most reliable marker for autophagy activation [33]. p62 protein can facilitate the degradation of poly-ubiquitinated proteins or organelles, and thus cause its own degradation. Therefore, decreased levels of p62 protein will induce the activation of autophagy and autophagic degradation [27]. Our western blot data indicates that EGCG treatment induces autophagic flux in HCT116 human colon cancer cells. Immunofluorescence staining confirms that EGCG treatment decreased the accumulation of p62 protein (Figure 3C). Collectively, these results demonstrate that EGCG induces autophagic flux in human colon cancer cells, rendering them resistant to TRAIL-induced apoptosis.

### Inhibition of autophagy sensitized TRAIL-induced apoptosis on EGCG treatment

**Figure 4:**
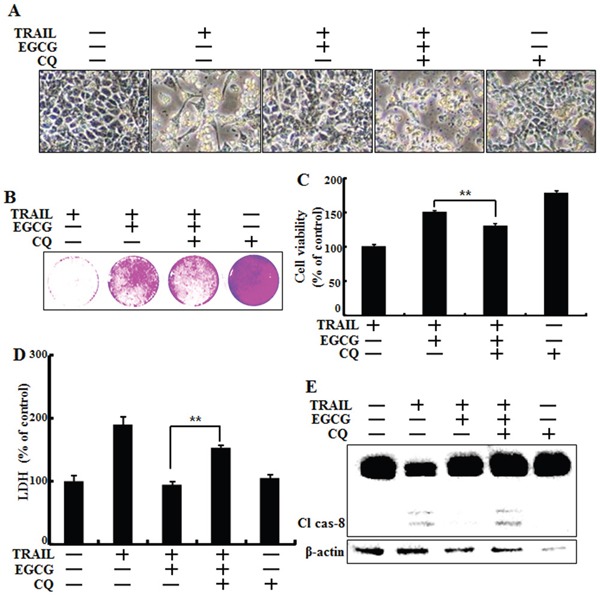
Inhibition of autophagy sensitized tumor cells to TRAIL-induced apoptosis on EGCG treatment HCT116 cells were pretreated with 50 nM chloroquine for 6 h, exposed to 20 μM EGCG for 12 h, and 100 ng/ml TRAIL for 2 h. **A.** Cell morphology was photographed under a light microscope (×200); **B, C.** Viability of treated cells was measured by crystal violet staining. The viability of control cells was considered as 100%; **D.** LDH released into the cell culture medium was measured after exposure to TRAIL for 3 h; **E.** Western blot analysis for cleaved caspase-8. β-actin was used as loading control; Bar graph indicating the total number of cells and the percentage of apoptotic cells. **p < 0.01 indicating significant differences between control group and treatment group.

Next, we examined the effect of a combined treatment of EGCG and chloroquine, a known autophagy inhibitor, on TRAIL treatment. HCT116 cells were pretreated with 50 nM chloroquine for 6 h and exposed to EGCG for 12 h. Cells were then treated with 100 ng/ml TRAIL for 2 h. Cell morphology, cell viability, and LDH release were monitoring using light microscopy and a crystal violet assay. Pharmacological inhibition of autophagy by chloroquine in the presence of EGCG sensitized HCT116 cells to TRAIL-induced cell death, compared to EGCG alone (Figure 4A-4D). The activated form of caspase-8, a known pro-apoptotic factor, was induced by chloroquine in cells co-treated with EGCG and TRAIL (Figure 4E). These data indicate that inhibition of autophagy increases TRAIL-related pro-apoptotic signaling in HCT116 cells.

### Inhibition of autophagic flux recovered the down-regulation of death receptors induced by EGCG treatment

**Figure 5:**
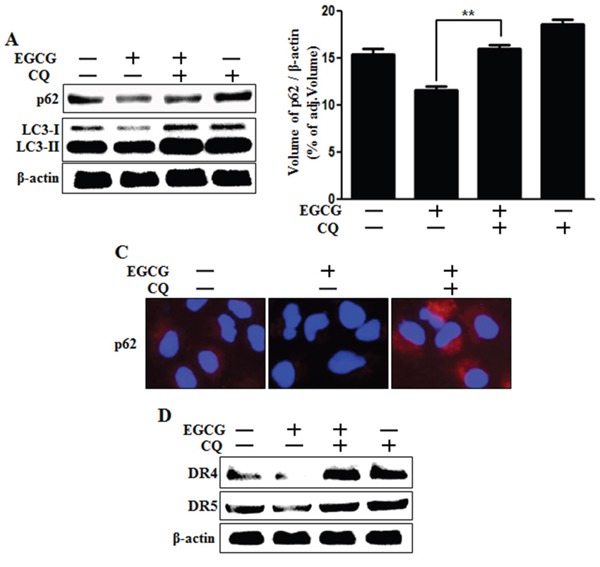
Inhibition of autophagic flux blocked the down-regulation of death receptors induced by EGCG treatment Cells were pretreated with 50 nM chloroquine for 6 h, exposed to 20 μM EGCG for 12 h. **A.** Cells subjected to western blot analysis for p62, LC3 proteins. β-actin was used as loading control; **B.** Bar graph indicating the averages of p62 protein levels. **C.** Representative images of p62 protein in HCT116 cells; **D.** Western blot analysis of death receptors DR4 and DR5. β-actin was used as loading control. **p < 0.01 indicating significant differences between control group and treatment group.

Next, we investigated whether death receptor DR4 and DR5 protein concentrations are correlated with inhibition of autophagic flux, by using western blot analysis. We confirmed that chloroquine could inhibit autophagic flux (Figure 5A-5C). Next, we examined the effect of chloroquine on death receptors. Western blot analysis data revealed that chloroquine increases the protein levels of death receptors (Figure 5D). These results demonstrate that autophagy can be inhibited by chloroquine-enhanced, TRAIL-induced apoptosis via death receptors.

### Genetic inhibition of autophagic flux promoted TRAIL-induced cell death upon EGCG treatment

**Figure 6:**
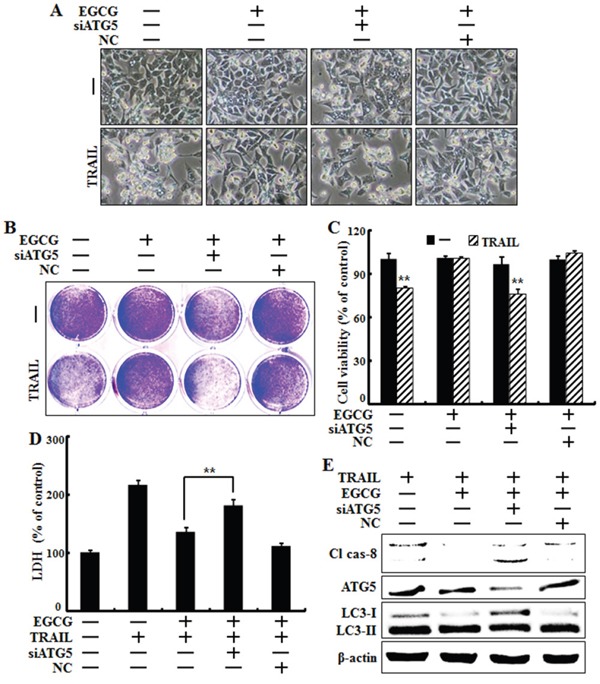
Genetic inhibition of autophagic flux promotes TRAIL-induced cell death upon EGCG treatment HCT116 cells were pre-treated with 20 nM ATG5 siRNA for 4 h, exposed to 20 μM EGCG for 12 h, and treated with 100 ng/ml TRAIL for 2 h. **A.** Cell morphology was photographed under a light microscope (×200); **B, C.** Viability of treated cells was measured by crystal violet staining. Viability of control cells was considered as 100%; **D.** LDH released into the cell culture medium was measured after exposure to TRAIL for 2 h; **E.** Western blot analysis of caspase-8, ATG5 and LC3. β-actin was used as loading control; **p < 0.01 indicating significant differences between control group and treatment group.

To determine whether EGCG-induced autophagy plays a protective role against TRAIL-induced apoptosis, we used ATG5 siRNA to study the effect of autophagy inhibition on TRAIL-induced apoptosis in HCT116 cells. The Atg12-Atg5-Atg16 complex is associated with the formation of the autophagosome, while LC3, also known as ATG8, is an autophagy marker lapidated during the induction of autophagic flux and is required for autophagosome formation [34, 35]. Cells were pretreated with 20 nM ATG5 siRNA for 24 h followed by treatment with EGCG for 12 h with or without co-treatment with 100 ng/ml TRAIL for 2 h. Based on cell morphology, ATG5 siRNA inhibited the effect of EGCG treatment on TRAIL-induced apoptosis (Figure 6A). The sensitivity of HCT116 cells to TRAIL-induced cell death was further studied using crystal violet and LDH assays. Our results show that ATG5 siRNA decreased cell viability but increased LDH levels (Figure 6B-6D). Functional knockdown of siRNA was confirmed by western blot data showing the down-regulation of ATG5 protein by ATG5 siRNA (Figure 6E). Next, we determined whether ATG5 siRNA increased the activation of caspase-8, our representative pro-apoptotic factor, using western blot analysis. The ATG5 siRNA in HCT116 cells increased the induction of cleaved caspase-8 after treatment with EGCG and TRAIL, and decreased the conversion of LC3-II protein (Figure 6E). These results demonstrate that genetic inhibition of autophagy by ATG5 siRNA enhances TRAIL-induced apoptosis and inhibits autophagic flux in HCT116 cells.

## DISCUSSION

TRAIL can induce apoptosis in various cancer cells. However, some cancer cells are not affected by TRAIL treatment [36]. The goal of this study was to overcome TRAIL-resistance by regulating death receptors and autophagic flux using EGCG. Our present study suggests that EGCG can activate autophagic flux in TRAIL-induced apoptosis by down-regulating death receptors and inhibiting TRAIL-induced mitochondrial dysfunction.

Recent studies have shown that EGCG treatment can protect against neurodegenerative disorders induced by prp106-126 by activating autophagic flux [16]. It has been demonstrated that autophagic flux is involved in TRAIL-induced apoptosis, and that the activation of autophagy can protect against TRAIL-induced cell death [19, 24]. However, the relationship between EGCG and an anti-cancer drug such as TRAIL has not been studied in any cancer cells until now.

Autophagy occurs in all nucleated type cells. It is a process essential to many cells, including animals, plants, and yeasts [37–39]. Activation of autophagy has been shown to prevent cell death in PCa cells, while inhibition of autophagy can enhance reagent-induced cell death [40, 41]. In this study, we showed that EGCG treatment activated autophagic flux and markedly decreased the protein levels of death receptors DR4 and DR5 (Figure 2 and Figure 3). To confirm that EGCG inhibited TRAIL-induced cell death via activation of autophagic flux, we used pharmacological (chloroquine) or genetic (ATG5-specific siRNA) inhibition of autophagy-sensitized TRAIL-induced cell death to compare with EGCG treatment alone (Figure 4 and Figure 6). Inhibition of autophagic flux was found to increase protein levels of death receptors compared to treatment with EGCG (Figure 5). These results indicate that EGCG might play an active role in cancer suppression, as it mediates the down-regulation of death receptors via activation of autophagic flux.

Many characteristics of autophagy activation are also exploited by cancer cells [28, 42]. Therefore, autophagy is an important target for cancer therapy. We found that EGCG treatment inhibited TRAIL-induced apoptosis and activated autophagic flux in HCT116 human colon cancer cells. Inhibition of autophagic flux by chloroquine and ATG5 siRNA antagonized the protective effect of EGCG. These results demonstrate that EGCG can protect against TRAIL-induced cell death by regulating autophagic flux in TRAIL-sensitive HCT116 cells. These observations collectively suggest that EGCG treatment might have beneficial side effects in anti-cancer therapy by activating autophagic flux and thus down-regulating death receptors. Therefore, we recommend that autophagy inhibitor be combined with another anti-cancer drug such as TRAIL in human cancer cells because of possible disturbances to the autophagic pathway.

## MATERIALS AND METHODS

### Cell culture

Human colon carcinoma cell line HCT116 was maintained in RPMI1640 medium containing 10% fetal bovine serum (FBS; Invitrogen-Gibco, Carlsbad, CA, USA) supplemented with 100 μg/ml penicillin-streptomycin in a humidified incubator maintained at 37°C with 5% CO_2_.

### Protein isolation and western blotting

Proteins were subjected to 10-15% sodium dodecyl sulfate-polyacrylamide gel electrophoresis, transferred to nitrocellulose membranes, and subjected to western blotting as described previously [43]. Antibodies used for immunoblotting were specific for Bax (Santa Cruz Biotechnology, Santa Cruz, CA, USA); caspase-8, p62, LC3, ATG5 (Cell Signaling Technology, Danvers, MA, USA), DR4, DR5, and β-actin (Sigma-Aldrich, St Louis, MO, USA).

### Crystal violet assay

Cells were seeded at 1×10^4^ cells/well into 12-well plates and incubated at 37°C for 24 h. Cells were pretreated with chloroquine for 6 h, EGCG for 12 h, and incubated with recombinant TRAIL for an additional 2 h. Cell morphology was examined using an inverted microscope (Nikon, Japan). Cell viability was determined using the crystal violet staining method as described previously [44]. Briefly, cells were stained for 10 min at room temperature with a staining solution (0.5% crystal violet in 30% ethanol and 3% formaldehyde), washed four times with water, and dried. Cells were then lysed with 1% SDS solution. Absorbance was measured at 550 nm. Cell viability was calculated based on relative dye intensity of treated cells compared to that of non-treated control cells.

### Lactate dehydrogenase assay

Cytotoxicity was assessed using cell supernatant and LDH Cytotoxicity Detection kit (Takara Bio, Tokyo, Japan) according to the manufacturer's protocol. LDH activity was determined based on absorbance at 490 nm.

### Immunofluorescence staining

Cells cultured on glass coverslips were treated with EGCG and chloroquine under normoxia. Cells were washed with PBS and fixed with cold acetone for 90 sec at room temperature. These cells were washed with PBS again, blocked with 5% fetal bovine serum in Tris-buffered saline with Tween 20, and incubated with monoclonal antibodies against p62 for 24 h at room temperature. Unbound antibody was removed by an additional wash with PBS. Cells were then incubated with anti-mouse Alexa Fluor 546 (for anti-p62) IgG antibody (4μg/ml) and anti-rabbit Alexa Flour 488 (for anti-LC3, DR5 and cleaved-caspase3) for 2 h at room temperature. Cells were then mounted with DakoCytomation medium and visualized using fluorescence microscope.

### RNA interference

HCT116 cells were transfected with ATG5-specific small interfering RNA (siRNA; Stealth RNAi, Santa Cruz Biotechnology) using Lipofectamine 2000 according to the manufacturer's instructions. Cells were plated into 24-well plates, pretreated with 20 nM ATG5 siRNA for 24 h, and incubated with recombinant TRAIL (0-100 ng/ml) for an additional 3 h under the same conditions. Scrambled ATG5 siRNA (Invitrogen) was used as negative control.

### Statistics

All data were expressed as means ± standard deviation (SD). Comparisons were performed using Student's t-test and ANOVA Duncan test with SAS statistical package (SAS Institute, Cary, NC, USA). Statistical significance was considered at *p* < 0.05 (*) or *p* < 0.01 (**).
